# Low Dietary Diversity for Recommended Food Groups Increases the Risk of Obesity among Children: Evidence from a Chinese Longitudinal Study

**DOI:** 10.3390/nu14194068

**Published:** 2022-09-30

**Authors:** Haiquan Xu, Songming Du, Ailing Liu, Qian Zhang, Guansheng Ma

**Affiliations:** 1 Institute of Food and Nutrition Development, Ministry of Agriculture and Rural Affairs, Beijing 100081, China; 2 Chinese Nutrition Society, Beijing 100022, China; 3 National Institute for Nutrition and Health, Chinese Center for Disease Control and Prevention, Beijing 100050, China; 4 Department of Nutrition and Food Hygiene, School of Public Health, Peking University, Beijing 100191, China

**Keywords:** dietary diversity scores, childhood obesity, Chinese children, longitudinal study

## Abstract

The association between dietary diversity and childhood obesity remains unclear; therefore, this longitudinal study was conducted to analyze the effect of dietary diversity on childhood obesity. One year after the first investigation, a follow-up was completed in 2010. A total of 4538 participants were included for analysis. Dietary diversity scores were calculated based on the consumption of nine recommended food groups which were categorized in accordance with the 2013 United Nations Food and Agriculture Organization guidelines. After a one-year follow-up, the low-score group underwent a significantly more considerable change in weight, body mass index, and body fat percentage than the high-score group (4.62 vs. 4.06 kg, 0.76 vs. 0.51 kg/m^2^, and 1.99% vs. 1.13%, respectively). Furthermore, in the low-score group, the odds ratios for overweight, obese, and overweight and obese were 1.76 (95% CI: 1.17, 2.65), 0.99 (95% CI: 0.67, 1.46), and 1.35 (95% CI: 1.01, 1.81), and the relative risks were 1.81 (95% CI: 1.03, 3.19), 2.31 (95% CI: 0.81, 6.59), and 1.98 (95% CI: 1.20,3.28), respectively. Low dietary diversity for the recommended food groups was associated with a high weight, high body mass index, and high body fat, which was associated with an increased risk of being overweight or obese in Chinese children.

## 1. Introduction

Obesity is a major risk factor for several diseases, and it has a significant economic influence on global healthcare systems [1,2,3]. Furthermore, childhood obesity can have a long-term influence on growth, development, and adult health, and it has become a major public health problem worldwide. Economic development and complex lifestyles have led to changes in dietary patterns and nutrition transitions that have rapidly increased the prevalence of childhood obesity in China. According to the Chinese Residents’ Nutrition and Chronic Disease Report (2020), the combined prevalence of overweight and obese children aged 6–17 years increased from 6.6% in 2002 to 19.0% in 2015–2017, and has thus attracted increasing attention [4,5]. If these trends continue, by 2030, the prevalence of overweight and obese children will reach 28.0%, and 30.6 and 18.9 million children will be overweight and obese, respectively [6]. Due to this prevalence in children, comprehensive action should be taken to improve children’s health.

Research has focused on the role of single nutrients in obesity [7]. In addition, dietary pattern analysis has been proposed as a novel approach for assessing the association between diet and the risk of chronic diseases, including obesity. Dietary pattern analysis involves investigating the effects of the overall diet rather than individual nutrients or foods, and this approach may thus be more indicative of disease risk than individual foods or nutrients [8,9]. The overall diet can be analyzed through the dietary diversity score (DDS), which has been used to evaluate nutrient adequacy and the quality of diets [10,11,12,13]. Research has demonstrated that a higher DDS is associated with higher diet quality [14]. The dietary guidelines of many countries list dietary diversity as being characteristic of a healthy diet [15,16]. The Chinese Dietary Guidelines (2022), published in 2022, also highlight the importance of dietary diversity and recommend that individuals consume at least 12 types of foods per day and 25 types per week [16]. Although some studies have focused on the association between dietary diversity and obesity, the results obtained are inconsistent among both adults and children [13,17,18,19,20,21]. Based on the existing evidence of observational studies, the American Heart Association pointed out that implementing greater dietary diversity could not be undertaken as an effective strategy to promote healthy eating patterns and a healthy body weight for adults in 2018; however, it is still uncertain for children.

Food can be categorized into groups according to multiple factors. Considering processed foods, refined grains, and sugar-sweetened beverages are not encouraged, in this study, only the recommended foods were categorized into nine groups in accordance with the Food and Agriculture Organization of the United Nations’ (FAO) guidelines and an average child’s diet in China. The association between dietary diversity for healthy food groups and childhood obesity was analyzed to explore the double burden of malnutrition for Chinese children who were born and grew up in a nutrition transition period.

## 2. Materials and Methods

### 2.1. Study Design

**Figure 1:**
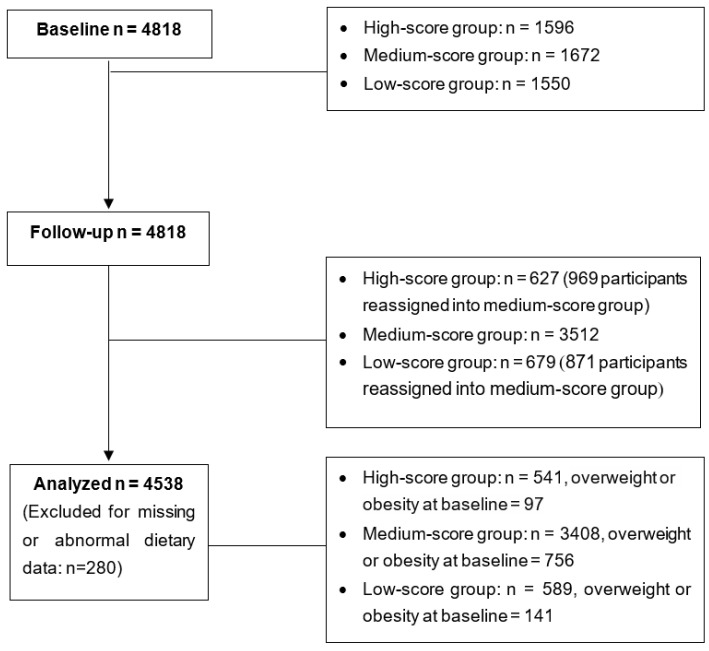
The study profile for data analysis.

This study used a convenience sample from the nutrition-based comprehensive intervention study on childhood obesity in China, which is a multi-center cluster randomized control trial. Children from Shanghai, Chongqing, Guangzhou, Jinan, Harbin, and Beijing were recruited. First, six schools from each city were selected and randomly sorted into 2 groups (3 for control and 3 for intervention) as a result of a lottery. In total, 30 primary schools were included in this trial. Second, two classes were selected from each grade (1st to 5th) in every sample school. Comprehensive interventions, including nutrition education and physical activity interventions, were implemented, though none were implemented in the control school. Detailed information regarding this trial has been reported [22]. This study design was longitudinal to enable analysis of the association between dietary diversity and childhood obesity. The participating children were divided into three groups according to their dietary diversity levels (low, medium, and high), and a year after the first investigation, a follow-up appointment was carried out. A total of 4818 children were included at the baseline, and they continued with the study up until the follow-up appointment; the data of 4538 participants with both anthropometric measurements and dietary intake data were included in the final analyses (Figure 1).

The protocol of this study was approved by the Ethics Committee of the National Institute of Nutrition and Food Safety, Chinese Center for Disease Control and Prevention (Code: 20081201) on 10 February 2009. Signed consent forms were obtained from both the parents and guardians and the children themselves.

### 2.2. Anthropometric Measurements

Physical examinations were completed at the participants’ schools. Height was measured to an accuracy of 1 mm with a free-standing stadiometer mounted on a rigid tripod (GMCS-I, Xindong Huateng Sports Equipment, Beijing, China). Overnight fasting body weight was measured to the nearest 0.1 kg on a digital scale (RGT-140, Wujin Hengqi, Changzhou, China). BMI was calculated as weight in kg divided by height in m^2^ (kg/m^2^). Overweight and obesity statuses were defined based on BMI in accordance with the Chinese national standard for screening overweight and obese school-age children and adolescents [23]. Waist circumference (WC) was measured mid-way between the lower rib margin and the iliac crest with a flexible anthropometric tape (Myotape). WC was measured twice to the nearest 0.1 cm. If the difference between two measurements was more than 0.5 cm, a third measurement was taken, and the mean was calculated using the two closest measurements. Body fat (BF) was measured in the morning by using a single-frequency (50 Hz) hand-to-foot bioelectrical impedance device (ImpDF50, ImpediMed, Queensland, Australia) after an overnight fast. The BF percentage was calculated using the prediction equations developed by Deurenberg [24].

### 2.3. Sociodemographic Information

Sociodemographic information was collected using a questionnaire. The parental education level was defined by the maternal education level when available. When the maternal education level was not available, the paternal education level was used as a surrogate. Each family’s income level was classified as monthly household income per capita in accordance with figures from 2009. Moreover, other information concerning family and participant characteristics was also collected, such as race, home environment, job, and so on.

### 2.4. Dietary Diversity Measurements

Information on dietary intake was collected through a self-administered 24 h dietary recall assessment, in which the participants recorded the food and drink they consumed for 3 consecutive days (2 weekdays and 1 weekend day), either by themselves or with their parents’ assistance. The children were taught how to complete the dietary records by trained research assistants. In accordance with the 2013 FAO guidelines, the food types were categorized as starchy staples (including cereals, roots, and tubers, but sweet biscuits and cakes were not included); dark-green leafy vegetables; vitamin A-rich fruits and vegetables; other fruits and vegetables; organ meat; meat and fish; eggs; legumes, nuts, and seeds; and milk and milk-based products [25]. Limited foods, such as sweets, snacks, and caloric beverages were not included in these groups. One point was assigned for each food category if the participant had consumed at least one food in the category. Daily scores were calculated through this method, the average of the three daily DDSs was used as the participant’s final score, and the outcome indicators were used for assessing dietary diversity. The participants were considered to have a diverse diet if they consumed nine types of foods daily. The three DDS groups were classified as follows: high-score group, both baseline DDS and follow-up DDs in the high tertile; low-score group, both baseline DDS and follow-up DDs in the low tertile; and medium-score group, which includes the rest.

### 2.5. Statistical Analysis

Continuous variables are expressed as mean and standard deviations, and categorical variables are expressed as rates and case numbers. The chi-square test and logistic regression were used to examine the association between the DDS and childhood obesity rate. The odds ratios (ORs) and relative risks (RRs) were used to demonstrate the association between the DDS and childhood obesity for the cross-sectional analysis (at baseline) and the longitudinal analysis, respectively. Sex, parental education level, family income level, and intervention were adjusted for the analysis. A linear mixed model, adjusted for potential confounding factors (weight status, sex, age, parental education level, and family income level), was used to compare the changes in continuous variables between the groups. The proportions were compared using a generalized linear mixed model. The significance was set to *p* < 0.05. Analyses were completed using SAS software, version 9.3 (SAS Institute, Cary, NC, USA).

## 3. Results

### 3.1. General Characteristics

A total of 4538 participants were included in the analyses (541, 3408, and 589 children in the high-, medium-, and low-score groups, respectively). The proportion of boys was 38.6%, 47.2%, and 59.8% in the high-, medium-, and low-score groups, respectively (*p* < 0.001). Significant differences in education and income levels were identified among the three groups. The DDSs for the high-, medium-, and low-score groups were 5.6 ± 0.6, 4.4 ± 1.0, and 3.0 ± 0.6 (*p* < 0.001) at baseline, and 5.5 ± 0.5, 4.2 ± 0.9, and 2.8 ± 0.5 (*p* < 0.001) at follow-up, respectively (Table 1).

### 3.2. Effect of Dietary Diversity on Weight, BMI, WC and BF

Compared with the high-score group, the weights, BMIs, and WCs in the medium- and low-score groups were significantly higher at baseline. After one year, greater increases in weight, BMI, and BF were identified in the low-score group compared with the high-score group, with the weights being 4.62 ± 4.92 and 4.06 ± 3.12 kg, BMIs being 0.76 ± 2.66 and 0.51 ± 1.34 kg/m^2^, and BFs being 1.99% ± 3.88% and 1.13% ± 3.23%, respectively (Table 2). A comparison of subgroups for the different sexes is shown in Supplementary Material Table S1.

### 3.3. Association between Dietary Diversity and Obesity

The prevalence of overweight children was 12.73%, 11.85%, and 7.39% (*p* = 0.005) in the low-, medium-, and high-score groups, respectively, and the prevalence of overweight and obese children was 23.94%, 22.23%, and 17.93% (*p* = 0.037), respectively (Table 3). The ORs of overweight children, obese children, and overweight and obese children in the low-score group were 1.76 (95% CI: 1.17, 2.65), 0.99 (95% CI: 0.67, 1.46), and 1.35 (95% CI: 1.01, 1.81), respectively, after adjustment for sex, education level, and income level (Table 4).

In the low-, medium-, and high-score groups, the incidence rates were 4.50%, 6.07%, and 8.71% for overweight children; 1.13%, 1.06%, and 3.13% for obese children; and 5.63%, 7.13%, and 11.84% for overweight and obese children at the follow-up appointment, respectively (Table 3). The RRs of overweight children, obese children, and overweight and obese children for the low-score group were 1.81 (95% CI: 1.03, 3.19), 2.31 (95% CI: 0.81, 6.59), and 1.98 (95% CI: 1.20, 3.28), respectively, after adjustment for sex, education level, income level, and intervention (Table 4).

## 4. Discussion

To the best of our knowledge, this is the first longitudinal study investigating the association between the dietary diversity of recommended food groups and childhood obesity among Chinese children. The results of this study revealed that low dietary diversity for recommended foods may be related to a high weight, BMI, and BF, which can significantly increase the risk of being overweight and obese. The OR (1.44, 95% CI: 1.08, 1.93) and RR (2.10, 95% CI: 1.33, 3.32) of overweight and obese children were significantly higher in the low-DDS group than in the high-DDS group.

Initially, dietary diversity was taken as an effective indicator of diet quality. As a higher DDS can reflect higher diet quality and a higher intake of nutrients, the DDS can be used as an indicator of diet quality in various countries. Consuming a wide variety of foods can ensure the adequate intake of essential nutrients and can lead to higher diet quality and more optimal health outcomes [26,27]. Additionally, dietary diversity has also been used as an indicator of health, some studies have focused on the association between dietary diversity, nutritional status, and health problems, such as obesity and micronutrient deficiency. Recently, an increasing number of studies showed that dietary diversity may increase energy intake and weight [28,29,30].

The results of relevant studies on children are inconsistent [13,31]. This may be due to the different methods for assessing dietary intake and determining DDSs [13]; therefore, well-designed prospective studies with similar approaches for assessing DDSs should be conducted. In this study, the dietary diversity was evaluated based on recommended food groups; limited foods, such as processed foods, refined grains, and sugar-sweetened beverages, were not included, and the results confirmed that high dietary diversity reduced the risk of childhood overweight or obesity. Therefore, we propose that higher dietary diversity based on the recommended food groups may lead to primary school children having a lower risk of being overweight and obese, in that it would require them to increase their daily healthy food intake frequency, develop more diverse dietary patterns, and increase consumption of the recommended food groups, thus helping to maintain an appropriate energy intake.

Compared with research in children, research in adults has revealed that a higher DDS may increase the risk of obesity [32]. Higher DDSs for all foods indicate the higher consumption of many food types, especially some processed or high energy-dense foods, which may cause excess energy intake and obesity in adults; this may explain why adults with a higher DDS may have a higher calorie intake and why they might be more obese [33,34]. Furthermore, evidence suggests that higher dietary diversity may be associated with suboptimal diet quality and higher food consumption and energy intake, particularly in middle-aged adults [28,29,30,35,36,37]. Foods with a lower glycemic load and high fiber content were reported to be more strongly and inversely associated with weight change than low-fiber foods and foods with a higher glycemic load [38]. Furthermore, in adults, studies reported a direct association between DDSs and energy intake, and they showed a higher energy intake, due to the higher consumption of sweets, snacks, condiments, entrées, and carbohydrates, coupled with a low variety of vegetables [39,40].

Although the findings regarding high dietary diversity showed an increased risk of energy intake and obesity for adults, some studies with dietary diversity scores for recommended foods indicated an inverse association between the DDS and obesity [19,40,41]. In 2018, a science advisory from the American Heart Association pointed out that greater dietary diversity was associated with greater energy intake, suboptimal eating patterns, and weight gain in adult populations. It is appropriate to promote a healthy eating pattern that emphasizes the adequate intake of plant foods, protein sources, low-fat dairy products, vegetable oils, and nuts, and limits the consumption of sweets, sugar-sweetened beverages, and red meats [42]; therefore, the public health programs should emphasize the diversity of recommended foods, rather than all foods, and limit unhealthy foods such as processed foods, refined grains, and sugar-sweetened beverages to promote healthy eating patterns and healthy body weight.

For children, we believe the recommendations from the American Heart Association for adults are still applicable, in that dietary diversity for recommended foods, but not processed foods or high energy dense foods, should be improved, and some interventions should be considered to achieve this goal. The results of this study indicated that comprehensive interventions, which include nutrition education, significantly affected the participants’ breakfast habits [43]. This study has several limitations. First, all of the participants were from urban areas, no children from rural areas were included. Moreover, this cohort is from a multicenter randomized control trial that does not represent a particular population; therefore, the generalizability of the study was limited. Second, the study period was short (only one year). The results may be more robust if a longer study period is used. Third, our findings may be limited due to residual confounding or confounding from unmeasured factors, such as physical activity, genetic factors, among others. Though intervention measures, including physical activity, was adjusted, the exact physical activity level is lacking. Lastly, although a dietary diversity assessment can reflect dietary quality, it cannot quantify the exact intake of various foods; therefore, if combined with the recommended servings, the results might be better.

## 5. Conclusions

This study demonstrated that low dietary diversity for recommended food groups is associated with a high weight, high BMI, and high BF, and is strongly associated with an increased risk of Chinese children being overweight and obese. Dietary diversity for recommended foods should be encouraged to improve dietary quality by ensuring adequate nutrient intake, balanced energy intake, and healthy growth. Attention should also be paid to limiting unhealthy foods such as processed foods, refined foods, and sugar-sweetened beverages; therefore, more attention should be paid to the dietary diversification of recommended foods as a preventive measure for childhood obesity, and this should become a dietary habit for children.

## Figures and Tables

**Table 1:** Participant characteristics.

	High-Score	Medium-Score	Low-Score	*p*
Sex (N (%))				
Boy	209 (38.6)	1610 (47.2)	352 (59.8)	<0.001
Girl	332 (61.4)	1798 (52.8)	237 (40.2)	
Group (N (%))				
Control	229 (42.4)	1455 (42.7)	267 (45.3)	0.494
Intervention	312 (57.6)	1953 (57.3)	322 (54.6)	
Education (N (%))				
Low	204 (39.2)	1491 (46.4)	295 (53.9)	<0.001
High	316 (60.8)	1725 (53.6)	252 (46.1)	
Income (N (%))				
Low	215 (41.3)	1374 (42.7)	298 (54.5)	<0.001
High	305 (58.6)	1842 (57.3)	249 (45.5)	
Age (year, Mean ± SD)	8.9 ± 1.2	9.0 ± 1.2	8.9 ± 1.2	0.004
DDS (Mean ± SD)				
Baseline (Mean ± SD)	5.6 ± 0.6	4.4 ± 1.0	3.0 ± 0.6	<0.001
Follow-up (Mean ± SD)	5.5 ± 0.5	4.2 ± 0.9	2.8 ± 0.5	<0.001

Comparison using the chi-square test and analysis of variance.

**Table 2:** Physical measurements at baseline and follow-up.

	High-Score	Medium-Score	Low-Score	*p*
Baseline				
Weight (kg, Mean ± SD)	31.49 ± 8.27	32.49 ± 8.85 *	32.79 ± 8.87 *	0.032
BMI (kg/m^2^, Mean ± SD)	16.74 ± 2.84	17.08 ± 3.13 *	17.45 ± 3.66 *	0.001
WC (cm, Mean ± SD)	57.00 ± 8.24	58.17 ± 8.69 *	58.96 ± 8.84 *	<0.001
BF (%, Mean ± SD)	27.73 ± 6.38	27.45 ± 6.66	27.02 ± 6.82	0.197
Follow-up (changes)				
Weight (kg, Mean ± SD)	4.06 ± 3.12	4.31 ± 3.78	4.62 ± 4.92 *	0.066
BMI (kg/m^2^, Mean ± SD)	0.51 ± 1.34	0.61 ± 1.69	0.76 ± 2.66 *	0.079
WC (cm, Mean ± SD)	3.12 ± 3.23	3.14 ± 3.54	3.13 ± 4.08	0.991
BF (%, Mean ± SD)	1.13 ± 3.23	1.35 ± 3.65	1.99 ± 3.88 *	0.001

* *p* < 0.05, compared with the high score group. The linear mixed model was used.

**Table 3:** Prevalence (baseline) and incidence rate (follow-up) of overweight and obese children (*n* [%]).

	High-Score	Medium-Score	Low-Score	*p*
Prevalence				
Sample size	541	3408	589	
Overweight	40 (7.39)	404 (11.85)	75 (12.73)	0.005
Obese	57 (10.54)	352 (10.38)	66 (11.21)	0.812
Overweight and obese	97 (17.93)	756 (22.23)	141 (23.94)	0.037
Incidence rate				
Sample size without overweight or obesity at baseline	444	2652	448	
Overweight	20 (4.50)	161 (6.07)	39 (8.71)	0.028
Obese	5 (1.13)	28 (1.06)	14 (3.13)	0.001
Overweight and obese	25 (5.63)	189 (7.13)	53 (11.84)	<0.001

The generalized linear mixed model was used.

**Table 4:** ORs (baseline) and RRs (follow-up) of overweight and obese children (OR [95% CI]/RR [95% CI]).

	High-Score	Medium-Score	Low-Score
ORs			
Model 1			
Overweight	1.00	1.68 (1.20, 2.36)	1.83 (1.22, 2.74)
Obese	1.00	0.98 (0.73, 1.32)	1.07 (0.74, 1.56)
Overweight and obese	1.00	1.31 (1.03, 1.65)	1.44 (1.08, 1.93)
Model 2			
Overweight	1.00	1.63 (1.16, 2.29)	1.68 (1.12, 2.52)
Obese	1.00	0.94 (0.7, 1.26)	0.97 (0.66, 1.41)
Overweight and obese	1.00	1.25 (0.99, 1.58)	1.30 (0.97, 1.74)
Model 3			
Overweight	1.00	1.73 (1.23, 2.44)	1.91 (1.27, 2.89)
Obese	1.00	0.99 (0.73, 1.33)	1.09 (0.75, 1.6)
Overweight and obese	1.00	1.33 (1.05, 1.69)	1.50 (1.11, 2.01)
Model 4			
Overweight	1.00	1.67 (1.19, 2.35)	1.84 (1.22, 2.76)
Obese	1.00	0.98 (0.72, 1.32)	1.08 (0.74, 1.58)
Overweight and obese	1.00	1.30 (1.03, 1.65)	1.46 (1.08, 1.96)
Model 5			
Overweight	1.00	1.66 (1.17, 2.34)	1.76 (1.17, 2.65)
Obese	1.00	0.94 (0.70, 1.28)	0.99 (0.67, 1.46)
Overweight and obese	1.00	1.27 (1.01, 1.61)	1.35 (1.01, 1.81)
RRs			
Model 6			
Overweight	1.00	1.35 (0.86, 2.12)	1.93 (1.15, 3.26)
Obese	1.00	0.94 (0.36, 2.42)	2.78 (1.01, 7.64)
Overweight and obese	1.00	1.27 (0.84, 1.90)	2.10 (1.33, 3.32)
Model 7	1.00		
Overweight	1.00	1.34 (0.85, 2.11)	1.94 (1.15, 3.27)
Obese	1.00	0.94 (0.36, 2.42)	2.82 (1.03, 7.77)
Overweight and obese	1.00	1.26 (0.84, 1.89)	2.12 (1.34, 3.34)
Model 8	1.00		
Overweight	1.00	1.30 (0.83, 2.06)	1.74 (1.01, 3.00)
Obese	1.00	0.89 (0.35, 2.32)	2.42 (0.81, 7.24)
Overweight and obese	1.00	1.22 (0.82, 1.83)	1.88 (1.16, 3.03)
Model 9	1.00		
Overweight	1.00	1.35 (0.86, 2.13)	1.75 (1.04, 2.95)
Obese	1.00	0.97 (0.37, 2.51)	2.94 (1.00, 8.69)
Overweight and obese	1.00	1.28 (0.85, 1.91)	1.97 (1.24, 3.12)
Model 10	1.00		
Overweight	1.00	1.35 (0.86, 2.12)	1.83 (1.10, 3.07)
Obese	1.00	0.93 (0.36, 2.40)	2.88 (1.00, 8.60)
Overweight and obese	1.00	1.26 (0.84, 1.89)	2.02 (1.28, 3.20)
Model 11	1.00		
Overweight	1.00	1.33 (0.82, 2.14)	1.81 (1.03, 3.19)
Obese	1.00	0.91 (0.35,2.39)	2.31 (0.81,6.59)
Overweight and obese	1.00	1.25 (0.81, 1.92)	1.98 (1.20, 3.28)

The generalized linear mixed model was used. Model 1: without adjustment; Model 2: adjusted for sex; Model 3: adjusted for education level; Model 4: adjusted for income level; Model 5: adjusted for sex, education level, and income level. Model 6: without adjustment; Model 7: adjustment for intervention; Model 8: adjustment for sex; Model 9: adjustment for education level; Model 10: adjustment for income level; Model 11: adjusted for sex, intervention, education level, and income level.

## Data Availability

The data presented in this study are available on request from the corresponding author. The data are not publicly available due to privacy or ethical restrictions.

## Supplementary Materials

The following supporting information can be downloaded at: https://www.mdpi.com/article/10.3390/nu14194068/s1, Table S1: The physical measurement at baseline and follow-up in subgroups.
