# Supplementary material for: Low Dietary Diversity for Recommended Food Groups Increases the Risk of Obesity among Children: Evidence from a Chinese Longitudinal Study
Source: Nutrients. 2022 Sep 30;14(19):4068. doi: 10.3390/nu14194068 (PMC9571432; doi:10.3390/nu14194068)
Supplement: Supplementary file 1 [file nutrients-14-04068-s001.zip › nutrients-1923953-supplementary.pdf]

Table S1. The physical measurement at baseline and follow-up in subgroups.

		High-score	Medium-score	Low-score	*p*
Boy	Baseline				
	Weight (kg, Mean±SD)	32.12±8.62	33.6±9.55	33.88±9.2	0.088
	BMI (kg/m^2, Mean±SD)	17.16±2.99	17.58±3.41	17.89±4.03*	0.067
	WC (cm, Mean±SD)	58.76±9.02	59.93±9.66	60.36±9.08	0.169
	BF (%, Mean±SD)	22.17±6.12	23.00±6.55	23.47±6.29*	0.084
	Follow-up (changes)				
	Weight (kg, Mean±SD)	3.91±3.2	4.4±4.04	4.63±5.75	0.193
	BMI (kg/m^2, Mean±SD)	0.49±1.42	0.66±1.81	0.78±3.26	0.318
	WC (cm, Mean±SD)	3.3±3.05	3.46±3.71	3.52±4.13	0.799
	BF (%, Mean±SD)	2.04±3.5	2.00±3.96	2.56±4.42	0.080
Girl	Bseline				
	Weight (kg, Mean±SD)	31.11±8.03	31.5±8.05	31.22±8.13	0.672
	BMI (kg/m^2, Mean±SD)	16.48±2.71	16.63±2.79	16.81±2.95	0.397
	WC (cm, Mean±SD)	55.9±7.53	56.61±7.4	56.93±8.1	0.219
	BF (%, Mean±SD)	31.12±3.51	31.38±3.55	32.16±3.46*	0.002
	Follow-up (changes)				
	Weight (kg, Mean±SD)	4.15±3.07	4.23±3.55	4.61±3.37	0.268
	BMI (kg/m^2, Mean±SD)	0.52±1.3	0.57±1.57	0.73±1.42	0.261
	WC (cm, Mean±SD)	3.01±3.33	2.86±3.35	2.55±3.94	0.300
	BF (%, Mean±SD)	0.57±2.93	0.78±3.26	1.18±2.74	0.093

*$p < 0.05$, compared with high score group.
